# Carnosine Decreases PMA-Induced Oxidative Stress and Inflammation in Murine Macrophages

**DOI:** 10.3390/antiox8080281

**Published:** 2019-08-06

**Authors:** Giuseppe Caruso, Claudia G. Fresta, Annamaria Fidilio, Fergal O’Donnell, Nicolò Musso, Giacomo Lazzarino, Margherita Grasso, Angela M. Amorini, Fabio Tascedda, Claudio Bucolo, Filippo Drago, Barbara Tavazzi, Giuseppe Lazzarino, Susan M. Lunte, Filippo Caraci

**Affiliations:** 1 Department of Laboratories, Oasi Research Institute—IRCCS, 94018 Troina, Italy; 2 Ralph N. Adams Institute for Bioanalytical Chemistry, University of Kansas, Lawrence, KS 66047-1620, USA; 3 Department of Pharmaceutical Chemistry, University of Kansas, Lawrence, KS 66047-1620, USA; 4 Department of Drug Sciences, University of Catania, 95125 Catania, Italy; 5 School of Biotechnology, Dublin City University, D09W6Y4 Dublin, Ireland; 6 Bio-Nanotech Research and Innovation Tower (BRIT), University of Catania, 95125 Catania, Italy; 7 Institute of Biochemistry and Clinical Biochemistry, Catholic University of Rome, 00168 Rome, Italy; 8 Fondazione Policlinico Universitario A. Gemelli IRCCS, Largo A. Gemelli 8, 00168 Rome, Italy; 9 Department of Biomedical and Biotechnological Sciences, University of Catania, 95125 Catania, Italy; 10 Department of Life Sciences, University of Modena and Reggio Emilia, 41125 Modena, Italy; 11 Center for Neuroscience and Neurotechnology, University of Modena and Reggio Emilia, 41125 Modena, Italy; 12 Department of Chemistry, University of Kansas, Lawrence, KS 66047-1620, USA

**Keywords:** carnosine, macrophages, superoxide, oxidative stress, inflammation, antioxidants

## Abstract

Carnosine is an endogenous dipeptide composed of β-alanine and L-histidine. This naturally occurring molecule is present at high concentrations in several mammalian excitable tissues such as muscles and brain, while it can be found at low concentrations in a few invertebrates. Carnosine has been shown to be involved in different cellular defense mechanisms including the inhibition of protein cross-linking, reactive oxygen and nitrogen species detoxification as well as the counteraction of inflammation. As a part of the immune response, macrophages are the primary cell type that is activated. These cells play a crucial role in many diseases associated with oxidative stress and inflammation, including atherosclerosis, diabetes, and neurodegenerative diseases. In the present study, carnosine was first tested for its ability to counteract oxidative stress. In our experimental model, represented by RAW 264.7 macrophages challenged with phorbol 12-myristate 13-acetate (PMA) and superoxide dismutase (SOD) inhibitors, carnosine was able to decrease the intracellular concentration of superoxide anions (O_2_^−^•) as well as the expression of Nox1 and Nox2 enzyme genes. This carnosine antioxidant activity was accompanied by the attenuation of the PMA-induced Akt phosphorylation, the down-regulation of TNF-α and IL-6 mRNAs, and the up-regulation of the expression of the anti-inflammatory mediators IL-4, IL-10, and TGF-β1. Additionally, when carnosine was used at the highest dose (20 mM), there was a generalized amelioration of the macrophage energy state, evaluated through the increase both in the total nucleoside triphosphate concentrations and the sum of the pool of intracellular nicotinic coenzymes. Finally, carnosine was able to decrease the oxidized (NADP^+^)/reduced (NADPH) ratio of nicotinamide adenine dinucleotide phosphate in a concentration dependent manner, indicating a strong inhibitory effect of this molecule towards the main source of reactive oxygen species in macrophages. Our data suggest a multimodal mechanism of action of carnosine underlying its beneficial effects on macrophage cells under oxidative stress and inflammation conditions.

## 1. Introduction

Reactive oxygen species (ROS) and reactive nitrogen species (RNS) are both part of natural aerobic cell metabolism and are involved in the pathophysiology of different diseases [1,2]. Under physiological conditions, the controlled production of ROS and RNS as well as the activity of the cell antioxidant machinery are well-balanced [3], otherwise a well-known phenomenon named “oxidative/nitrosative stress” will occur [4,5]. ROS, in most healthy cells, are formed as superoxide anions (O_2_^−^•) by mitochondria, that generates O_2_^−^• during the tetravalent reduction of molecular oxygen to water catalyzed by the electron transport chain and connected to adenosine triphosphate (ATP) production [2,6,7,8]. Spontaneous O_2_^−^• dismutation is remarkably accelerated by superoxide dismutase (SOD) activity, that leads to the formation of hydrogen peroxide and diatomic oxygen [9]. At physiological levels, O_2_^−^• is implicated in several functions such as the messenger of endothelial function [10] and in the phosphorylation and activation of numerous protein kinases [11]. When O_2_^−^• is overproduced and exceeds cell antioxidant defences, it can either generate secondary, more dangerous ROS, or can easily react with nitric oxide (NO) to form the so-called “deadly cocktail”, as defined by Estévez and Jordán [12]. Oxidative/nitrosative stress leads to the production of ROS and RNS, including hydroxyl radicals and peroxynitrite, which are able to damage fatty acids, proteins, DNA, carbohydrates, and mitochondria [12]. The reaction between O_2_^−^• and NO is faster than the detoxification reaction catalyzed by SOD [13]. ROS and RNS are widely recognized as important stress mediators of pro-inflammatory conditions, thus connecting oxidative stress and inflammation phenomena [14].

When inflammation processes take place, inducible nitric oxide synthase (iNOS), responsible for NO production [15], as well as NADPH oxidase (Nox), responsible for O_2_^−^• production [16], are overexpressed in immune cells such as macrophages and microglia [17,18]. Different types of cells are involved in the immune response, among others macrophages are those primarily activated [19,20]. Macrophages can be classified in two different and functionally distinct sub-populations: pro-inflammatory or classically-activated macrophages (M1) and anti-inflammatory or alternatively activated macrophages (M2) [21]. While the M1 sub-population is associated with a high production of pro-inflammatory cytokines (e.g., IL-1β, IL-6, TNF-α, and IL-1), ROS, and RNS, the M2 sub-population produces anti-inflammatory cytokines (e.g., IL-4, IL-10, IL-13, and TGF-β1) [22]. Both peripheral macrophages and microglia, the resident macrophages of the brain, play a pivotal role in several diseases characterized by both oxidative stress and inflammation [23,24].

Carnosine is a natural dipeptide (β-alanyl-L-histidine) discovered more than 100 years ago [25]. This molecule and its related compounds such as anserine (L-alanyl-1-methyl-L-histidine) and homocarnosine (γ-amino-butyryl-L-histidine) are widely distributed in mammalian tissues [26] and in a few number of invertebrates [27,28]. Carnosine is synthesized starting from β-alanine and L-histidine via the activity of the enzyme carnosine synthetase 1, through an ATP-consuming process [29,30]. Although carnosine is mainly found in muscles [31], where it can reach a concentration close to 20 mM [32], its levels in the brain may also be considered reasonably high, ranging from 0.7 to 2.0 mM [33]. There are different in vivo studies in the literature showing the ability of carnosine to protect against pathologies characterized by oxidative stress and/or inflammation. In the study carried out by Zhang et al., employing a neonatal rat model, carnosine was able to protect against hypoxia-ischemia brain damage [34]; Albrecht et al. demonstrated that carnosine treatment improves glucose metabolism, albuminuria, and pathology in BTBR ob/ob mice [35]; very recently, Bermúdez and colleagues carried out an in vivo study in which intranasal carnosine administration attenuated transcriptomic alterations and improved mitochondrial function in the Thy1-aSyn mouse model of Parkinson’s disease (PD) [36]; lastly, carnosine rescued cognitive deficits and decreased expression of the receptor for advanced glycation end products in blood vessels and microglial activation in a transgenic mouse model of Alzheimer disease (AD) fed with a high fat diet [37]. Most of carnosine protective activities are attributable to its antioxidant and anti-inflammatory properties [38]. The interaction of carnosine with specific receptors localized on the cell membrane has been shown to modulate macrophage function [39] by increasing their phagocytotic activity [40]. The ability of this dipeptide to modulate NO production and macrophages polarization has also been proved [41,42]. Very recently, carnosine has been shown to decrease both oxidative stress and inflammation in an in vitro model of amyloid-induced inflammation [43].

In the present study, we first investigated the production of O_2_^−^• induced by phorbol 12-myristate 13-acetate (PMA) and SOD inhibitors, in the absence or in the presence of increasing concentrations of carnosine and anserine, in RAW 264.7 cells, representing an established in vitro experimental model for studying oxidative stress, with particular reference to O_2_^−^• production [1,44,45,46,47]. Additionally, in order to understand the molecular mechanisms underlying the ability of carnosine in counteracting O_2_^−^• production, we studied the variation of parameters representative of cellular energy metabolism, the expression of oxidative stress-related enzymes, and the expression of pro- and anti-inflammatory cytokines in RAW 264.7 cells challenged with PMA and SOD inhibitors in the absence or in the presence of carnosine. Lastly, relying on the fact that Akt protein is overactivated in macrophages under pro-oxidative and pro-inflammatory conditions [48], we also evaluated the effect of carnosine on the PMA-induced activation of Akt in RAW 264.7 macrophages.

## 2. Materials and Methods 

### 2.1. Materials and Reagents

RAW 264.7 cells (ATCC^®^ TIB-71™), Dulbecco’s Modified Eagle Medium (DMEM), fetal bovine serum (FBS), and penicillin/streptomycin antibiotic solution were purchased from American Type Culture Collection (ATCC, Manassas, VA, USA). Ultrapure standards for high-performance liquid chromatography (HPLC), tetrabutylammonium hydroxide, L-carnosine, L-Anserine nitrate salt, anhydrous dimethyl sulfoxide (DMSO), potassium chloride, sodium dodecyl sulfate (SDS), phosphate-buffered saline (PBS), trypan blue solution, 2-methoxyestradiol (2-ME), cocktail of protease inhibitors, serine/threonine phosphatase inhibitors, tyrosine protein phosphatases inhibitors, RIPA buffer, bovine serum albumin, Bradford reagent, sodium diethyldithiocarbamate trihydrate (DDC), and PMA were all supplied by Sigma-Aldrich (St. Louis, MO, USA). Centrifuge tubes equipped with 3 kDa molecular weight cut-off filters and water, methanol, far-UV acetonitrile, and chloroform (all HPLC-grade) were supplied by VWR International (West Chester, PA, USA). MitoSOX Red probe was purchased from Life Technologies (Carlsbad, CA, USA). C-Chip disposable hemocytometer was purchased from Bulldog Bio, Inc. (Portsmouth, NH, USA). SuperScript III First-Strand Synthesis SuperMix, TE buffer, 25 and 75 mL polystyrene culture flasks, 60 × 15 mm polystyrene Petri dishes, phenol red-free RPMI-1640 medium, boric acid, sodium hydroxide, acetone, 2-propanol, ethanol (95%), Pierce^TM^ BCA Protein Assay Kit, and Ne-PER Nuclear and Cytoplasmic Extraction Reagents were obtained from Thermo Fisher Scientific Inc. (Pittsburgh, PA, USA). QuantiTect SYBR Green PCR Kits, RNeasy Mini Kit, and QuantiTect Primer Assays, were purchased from Qiagen (Hilden, Germany). Anti-GAPDH primary antibody was obtained from Millipore (Burlington, MA, USA); anti-nuclear factor erythroid 2-related factor 2 (Nrf2), anti-Akt, and anti-p(ser473)Akt primary antibodies were all obtained from Cell Signaling Technology Inc. (Danvers, MA, USA); anti-heme oxygenase 1 (HO-1) and anti-Histone H3 primary antibodies were purchased from Abcam (Cambridge, UK). Secondary goat anti-rabbit labeled with IRDye 680 and goat anti-mouse labeled with IRDye 800 were purchased from Li-COR Biosciences (Lincoln, NE, USA). 384-well plates were obtained by Roche Molecular Systems Inc. (Pleasanton, CA, USA). Eppendorf LoBind 1.5 mL Microcentrifuge Tubes PCR Clean as well as PCR tubes were both obtained from Eppendorf (Hamburg, Germany). Sylgard 184 polydimethylsiloxane (PDMS) prepolymer and curing agent were obtained from Ellsworth Adhesives (Germantown, WI, USA).

### 2.2. Cell Culture and Preparation

RAW 264.7 macrophages were cultured in DMEM plus supplements and maintained in polystyrene culture flasks as previously described in detail [1]. Cells were passaged every 2 to 3 days depending on the cell confluence to avoid overgrowth. On the day of the experiment, cells were harvested, 100 μL of the cell suspension was used for cell counting (C-Chip disposable hemocytometer), and plated in polystyrene culture flasks or Petri dishes at the appropriate density.

### 2.3. Superoxide Anion Levels Determination Using Microchip Electrophoresis with Laser-Induced Fluorescence (ME-LIF) and MitoSOX Red Probe

MitoSOX Red (5 mM), DDC (100 mM), 2-ME (16.5 mM), and PMA (1 mg/mL) stock solutions were prepared as previously described [47]. Macrophages, plated at the density of 3 × 10^6^ cells/flask and ready for the treatment, were incubated for 1 h with the two well-known SOD inhibitors, the cytosolic DDC (1 mM final concentration) and the mitochondrial 2-ME (50 μM final concentration), in presence of the MitoSOX Red probe (10 μM final concentration). Cells were then subjected to an acute (30 or 60 min) stimulation with PMA (1 μg/mL) to increase O_2_^−^• production. To investigate the effects of carnosine or its methylated analogue anserine on O_2_^−^• production, cells were pre-incubated for 24 h with increasing carnosine or anserine concentrations (5, 10, or 20 mM), before PMA stimulation. During the incubation of the macrophages with MitoSOX Red, the flasks were always covered with aluminium foil to minimize any photobleaching of the probe. Untreated cells from the same population were used as a control. Cells were then harvested, counted, centrifuged, and the obtained cell pellet was prepared and analyzed by ME-LIF as recently described [43].

Supplementary Figure S1 depicts the experimental procedure to measure O_2_^−^• production in RAW 264.7 macrophages.

### 2.4. HPLC Analysis of Metabolites

Macrophage cells, plated at the density of 4.5 × 10^6^ cells/Petri dish and stimulated as described in Section 2.3, were deproteinized according to the organic solvent deproteinization, suitable to measure acid labile and easily oxidizable compounds described in detail elsewhere [49]. The analytes of interest were separated and quantified by ion-pairing HPLC method as previously described in detail [50,51]. Identification and measurement of the different compounds in chromatographic runs of cell extracts were performed by comparing retention times, absorption spectra, and area of the peaks of chromatographic runs of mixtures containing known concentrations of true ultrapure standard mixtures.

### 2.5. Gene Expression Analysis by Quantitative Real-Time PCR (qRT-PCR)

The concentration of total RNA recovered from 4.5 × 10^6^ cells (previously seeded in Petri dishes) by using RNeasy Mini Kit was determined by measuring the absorbance at 260 nm with a NanoDrop^®^ ND-1000 (Thermo Fisher Scientific, Waltham, MA, USA). The reverse transcription of 100 ng of total RNA (for each sample) was accomplished by using the SuperScript III First-Strand Synthesis SuperMix kit, while the quantification of each cDNA sample loaded in a 384-well plate was achieved by using a LightCycler^®^ 480 System (Roche Molecular Systems, Inc., Pleasanton, CA, USA). The list containing the information of each primer used for this study is reported in Table 1.

The protocol employed for sample amplification, fluorescence data collection, and sample quantification is the same as previously described by us [43].

### 2.6. Western Blot Analysis

Western blot analyses were performed as previously described [54] on macrophages harvested at 4 °C in RIPA buffer in the presence of a cocktail of protease inhibitors, serine/threonine phosphatase inhibitors, and tyrosine protein phosphatases inhibitors. After a sonication step, each lysate was subjected to centrifugation and supernatant collection. The protein concentrations were determined by Bradford reagent and by measuring the absorbance at 595 nm with a Varioskan^®^ Flash spectrophotometer (Thermo Fisher Scientific, Waltham, MA, USA). A standard curve was made by using bovine serum albumin. After blocking, membranes were incubated with the selected primary antibodies (1:2000 for GAPDH; 1:500 for all other primary antibodies) overnight at 4 °C. After 3 washing steps, membranes were incubated at room temperature, 60 min, with secondary goat anti-rabbit labeled with IRDye 680 and goat anti-mouse labeled with IRDye 800 (1:15000 for both of them). 

The nuclear translocation of Nrf2 in macrophages under all our experimental conditions was measured by employing Ne-PER Nuclear and Cytoplasmic Extraction Reagents following the manufacturer’s specification. Briefly, after centrifugation at 500× *g* for 3 min, the cellular pellet was re-suspended in ice-cold CER I, vortexed, and incubated on ice. Next, the suspension was added of ice-cold CER II and centrifuged in order to obtain supernatant cytoplasmic extract. The residual pellet (nuclear fraction) was suspended in ice-cold NER, incubated on ice, and vortexed several times. Lastly, the samples were centrifuged to obtain supernatant nuclear extract. After protein concentration determination by using Pierce^TM^ BCA Protein Assay Kit, nuclear samples were analyzed as described in the case of total protein extracts (Akt, pAkt, Nrf2, and HO-1). The dilution of Histone H3, used to normalize the densitometric values of nuclear Nrf2 [55], was 1:30000.

Hybridization signals were detected with the Odyssey Infrared Imaging System (LI-COR Biosciences, Lincoln, NE, USA). Western blot data were quantified by densitometric analysis (Image J software) of the hybridization signals.

### 2.7. Statistical Analysis

Graphpad prism (Graphpad software, San Diego, CA, USA) was the software selected to perform the statistical analysis. Normal data distribution was tested using the Jarque–Bera test, before performing the comparison within groups using the one-way analysis of variance (ANOVA) and the Tukey test for multiple comparisons, as the post hoc test. Only two-tailed *p*-values less than 0.05 were considered statistically significant.

## 3. Results

### 3.1. Carnosine Effects on PMA-Induced O_2_^−^• Production in Cultured Macrophages

Before monitoring the effects of carnosine or its methylated analogue anserine (5, 10, or 20 mM, indicated in each figure as C5, C10, C20, or A5, A10, A20, respectively), we first investigated the effects of acute stimulations of RAW 264.7 macrophages with 1 µg/mL PMA for 30 or 60 min on O_2_^−^• production.

**Figure 1:**
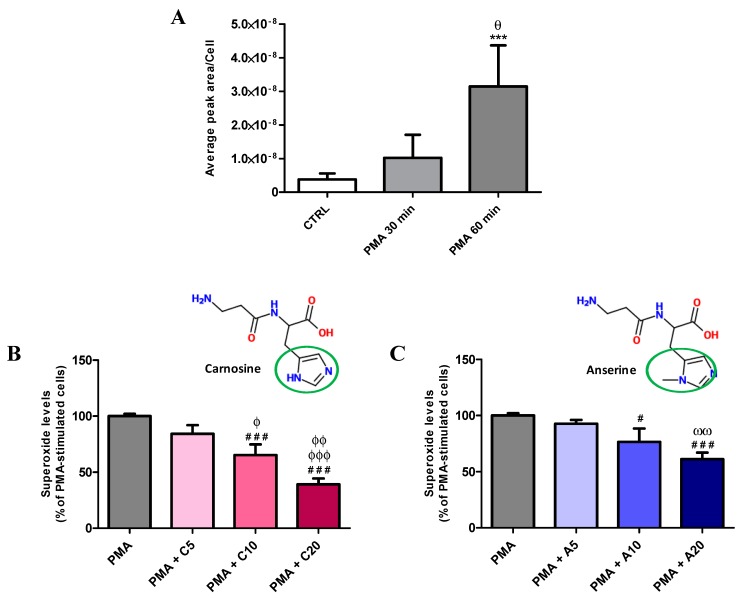
Detection of intracellular concentrations of O_2_^−^• (as detected by microchip electrophoresis with laser-induced fluorescence (ME-LIF)) in resting (control) macrophages and in macrophages stimulated with phorbol 12-myristate 13-acetate (PMA) and superoxide dismutase (SOD) inhibitors, in absence or presence of increasing concentrations (5, 10, or 20) of carnosine or anserine. (**A**) Change in O_2_^−^• production (expressed as Average peak area/Cell) in macrophages subjected to two different acute stimulations with PMA. (**B**,**C**) show the ability of carnosine and anserine in decreasing the intracellular concentrations of O_2_^−^• PMA-induced, respectively. The O_2_^−^• levels are expressed as the percent variation with respect to the PMA-stimulated cells. Values are means ± SD of three to five independent experiments. *** *p* < 0.001 vs. CTRL; ^θ^ *p* < 0.05 vs. PMA 30 min; ^#^ *p* < 0.05 vs. PMA; ^###^ *p* < 0.001 vs. PMA; ^ϕ^ *p* < 0.05 vs. PMA + C5; ^ϕϕ^ *p* < 0.01 vs. PMA + C10; ^ϕϕϕ^ *p* < 0.001 vs. PMA + C5; ^ωω^ *p* < 0.01 vs. PMA + A5.

Figure 1A clearly shows the time-dependent increase in O_2_^−^• production induced by acute stimulation with PMA. The shorter stimulation time (30 min) lead to a ~2.7-fold increase in O_2_^−^• production compared to control (untreated) cells, while cells stimulated for the longer time (60 min) had a further remarkable increase in O_2_^−^• production compared to both 30-min stimulation (~3.1-fold increase, *p* < 0.01) and controls (~8.3-fold increase, *p* < 0.001).

Once the optimal cell stimulation protocol was established (60 min stimulation with 1 µg/mL PMA), the effects of increasing carnosine and anserine (5, 10, or 20 mM) on intracellular O_2_^−^• levels in stimulated macrophages were tested. Carnosine decreased O_2_^−^• production in PMA-stimulated macrophages in a concentration-dependent manner (Figure 1B). Unlike 5 mM carnosine (−13% in O_2_^−^• production, not significant), the pre-treatment with 10 mM (−30%, *p* < 0.001 vs. PMA; −17%, *p* < 0.05 vs. PMA + C5) or 20 mM carnosine (−62%, *p* < 0.001 vs. PMA; −49%, *p* < 0.001 vs. PMA + C5; −32%, *p* < 0.01 vs. PMA + C10) led to significant decrease of intracellular O_2_^−^•. To evaluate the impact of the freedom of the imidazole ring on the decrease of O_2_^−^• levels we have replicated the experiment using anserine instead of carnosine. As observed for carnosine, the pre-treatment with anserine decreased O_2_^−^• production in PMA-stimulated RAW 264.7 macrophages (Figure 1C). The pre-treatment with 5 mM anserine did not affect the O_2_^−^• levels (−8%, not significative), while both 10 mM (−24%, *p* < 0.05 vs. PMA) and 20 mM (−39%, *p* < 0.001 vs. PMA; −31%, *p* < 0.01 vs. PMA + A5) significantly decreased O_2_^−^• levels. It is however worth underlining that each anserine concentration tested was less effective than the corresponding concentration of carnosine. In particular, 20 mM carnosine caused 1.6 times stronger decrease in O_2_^−^• levels (−62%) compared to the same concentration of its methylated analogue anserine (−39%, *p* < 0.01 compared to 20 mM carnosine).

### 3.2. Influence of Carnosine on Energy Metabolism of Cultured Macrophages

**Figure 2:**
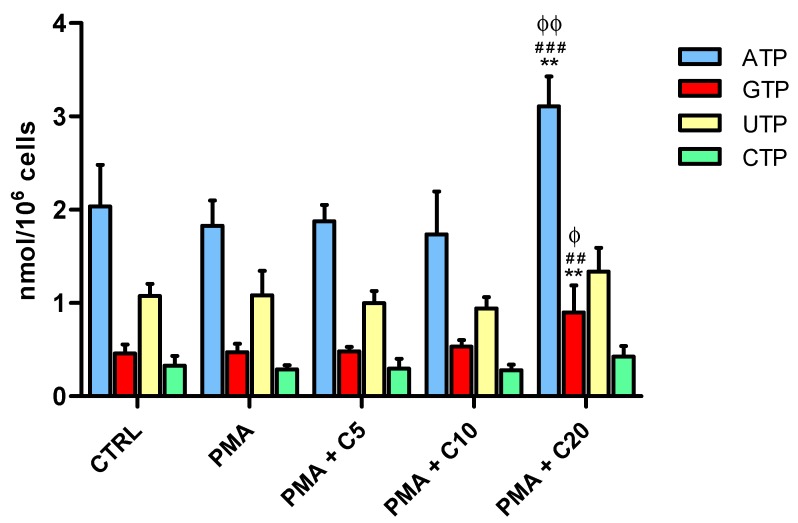
Changes in nucleoside triphosphate concentrations (ATP, GTP, UTP, and CTP), expressed as nmol/million cells, in resting (control) macrophages and in macrophages stimulated with PMA and SOD inhibitors, in absence or presence of increasing concentrations (5, 10, or 20) of carnosine. Values are means ± SD of four independent experiments. ** *p* < 0.01 vs. CTRL; ^##^ *p* < 0.01 vs. PMA; ^###^ *p* < 0.001 vs. PMA; ^ϕ^ *p* < 0.05 vs. PMA + C5 and PMA + C10; ^ϕϕ^ *p* < 0.01 vs. PMA + C5 and PMA + C10.

In Figure 2, the results show the effects of PMA and SOD inhibitors in absence or presence of increasing concentrations of carnosine (5, 10, or 20 mM) on energy metabolites concentrations (ATP, GTP, UTP, and CTP) of RAW 264.7 macrophages.

The short incubation time with PMA did not cause changes in any of these compounds. Similarly, 5 or 10 mM carnosine had no effects on the nucleoside triphosphates considered. Interestingly, 24 h incubation with 20 mM carnosine before PMA challenge induced a significant increase in both ATP (3.10 ± 0.32 nmol/10^6^ cells, *p* < 0.01 vs. CTRL; *p* < 0.001 vs. PMA; *p* < 0.01 vs. PMA + C5 or C10) and GTP (0.89 ± 0.29 nmol/10^6^ cells, *p* < 0.01 vs. CTRL; *p* < 0.01 vs. PMA; *p* < 0.05 vs. PMA + C5 or C10), while no effect was observed for UTP (1.33 ± 0.25 nmol/10^6^ cells) and CTP (0.42 ± 0.11 nmol/10^6^ cells). The total amount of nucleoside triphosphates (ATP + GTP + UTP + CTP) was 3.89 nmol/10^6^ cells in control (untreated) cells and increased up to 5.76 nmol/10^6^ cells (+47.9%) in PMA + 20 mM carnosine-treated cells. Overall these data show a reinforcement of the cellular phosphorylating capacity following the addition of 20 mM carnosine to macrophages before PMA challenge.

**Figure 3:**
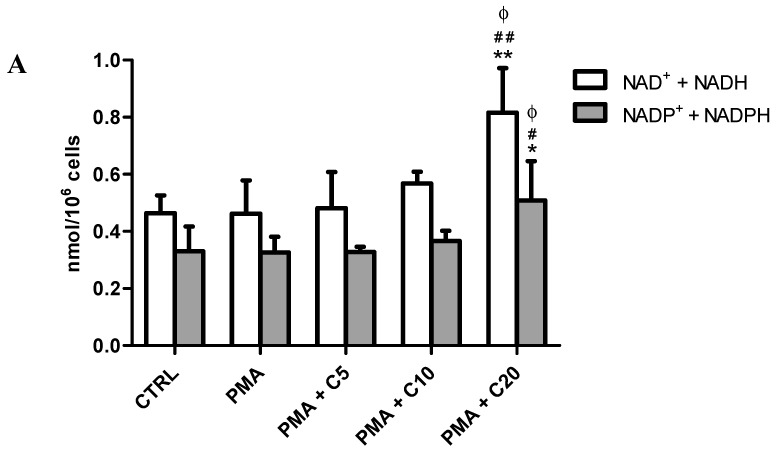

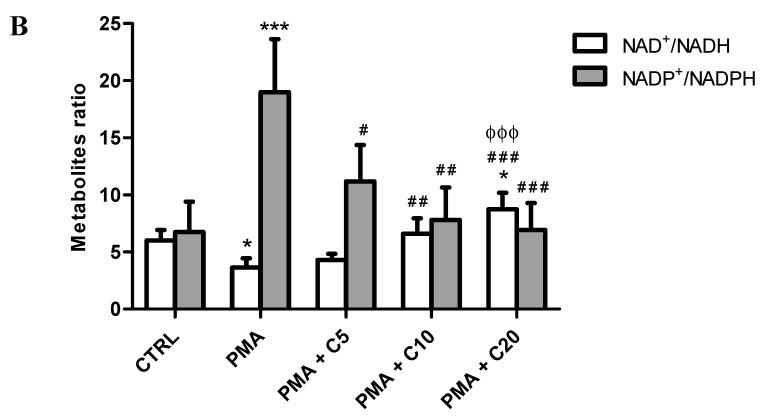
Consequences of the different cell treatments on (**A**) the sum (NAD^+^ + NADH and NADP^+^ + NADPH) and (**B**) the ratio (NAD^+^/NADH and NADP^+^/NADPH) of intracellular nicotinic coenzymes in resting (control) macrophages and in macrophages stimulated with PMA and SOD inhibitors, in absence or presence of increasing concentrations (5, 10, or 20) of carnosine. The sum of intracellular nicotinic coenzymes is expressed as nmol/million cells. Values are means ± SD of four independent experiments. * *p* < 0.05 vs. CTRL; ** *p* < 0.01 vs. CTRL; *** *p* < 0.001 vs. CTRL; ^#^ *p* < 0.05 vs. PMA; ^##^ *p* < 0.01 vs. PMA; ^###^ *p* < 0.001 vs. PMA; ^ϕ^ *p* < 0.05 vs. PMA + C5 and PMA + C10; ^ϕϕϕ^ *p* < 0.001 vs. PMA + C5.

In the same experimental conditions, we evaluated the levels of intracellular nicotinic coenzymes (NAD^+^ + NADH and NADP^+^ + NADPH) (Figure 3A).

The sum of NAD^+^ + NADH, as well as that of NADP^+^ + NADPH, were not significantly affected at any experimental conditions excepted for the PMA + C20 treatment. Indeed, significant increases in both NAD^+^ + NADH (0.82 ± 0.16 nmol/10^6^ cells, *p* < 0.01 vs. CTRL; *p* < 0.01 vs. PMA; *p* < 0.05 vs. PMA + C5 or C10) and NADP^+^ + NADPH (0.51 ± 0.01 nmol/10^6^ cells, *p* < 0.05 vs. all other treatments) were observed when using PMA + C20 treatment, indicating a better coenzymatic set for oxido-reductive reactions. The apparent lack of effects of PMA was vice versa manifested when considering the NAD^+^/NADH and NADP^+^/NADPH ratio (Figure 3B). PMA caused a 1.65-folds decrease of NAD^+^/NADH (*p* < 0.05 vs. CTRL) that did not ameliorate with 5 mM carnosine. Differently, 10 mM carnosine normalized this ratio (not significant vs. CTRL; *p* < 0.01 vs. PMA) and 20 mM carnosine caused a further increase up to values 1.45- and 2.40-folds higher than control and PMA, respectively (*p* < 0.05 vs. CTRL; *p* < 0.001 vs. PMA; *p* < 0.001 vs. PMA + C5). A different trend was observed when considering the NADP^+^/NADPH ratio; a 2.81-folds increase was measured for cells treated with PMA and SOD inhibitors only (*p* < 0.001 vs. CTRL). A dose-response effect was noticed for carnosine treatment, which, at 10 and 20 mM concentrations, normalized the ratio of these nicotinic coenzymes (not significant vs. CTRL; *p* < 0.01 vs. PMA).

### 3.3. Carnosine Effects on PMA-Induced Inflammatory Response of Cultured Macrophages

**Figure 4:**
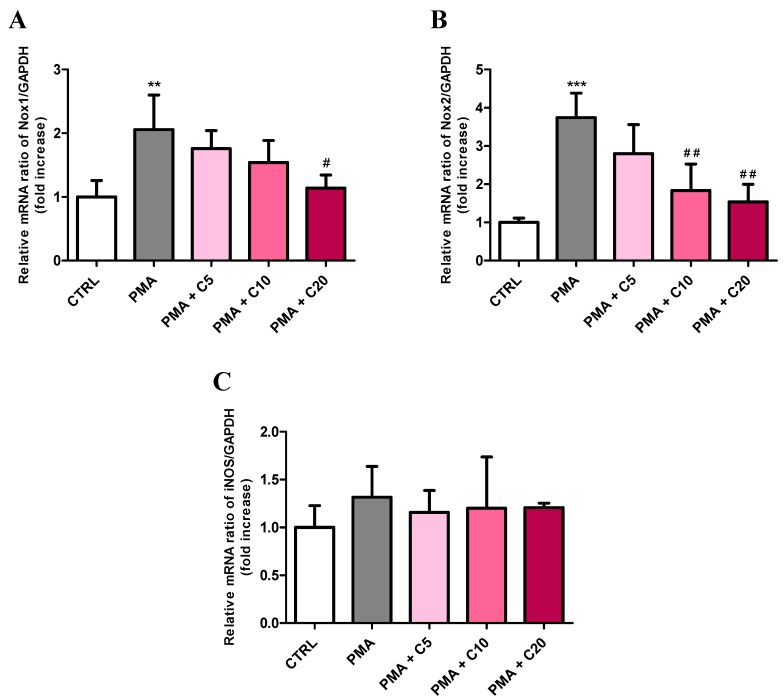
Measurement of (**A**) Nox1, (**B**) Nox2, and (**C**) iNOS mRNA expression levels (quantitative real-time PCR (qRT-PCR)) in resting (control) macrophages and in macrophages stimulated with PMA and SOD inhibitors, in absence or presence of increasing concentrations (5, 10, or 20) of carnosine. The abundance of each mRNA of interest was expressed relative to the abundance of GAPDH-mRNA, as an internal control. As a negative control, a reaction in absence of cDNA (no template control, NTC) was performed. Values are means ± SD of three to seven independent experiments. ** *p* < 0.01 vs. CTRL; *** *p* < 0.001 vs. CTRL; ^#^ *p* < 0.05 vs. PMA; ^##^ *p* < 0.01 vs. PMA.

Since the treatment of RAW 264.7 macrophages with carnosine decreased the PMA-induced O_2_^−^• production (Figure 1B), we assessed the ability of carnosine to modulate the expression of Nox1 and Nox2 as well as that of iNOS enzyme and the genes responsible for the transcription of pro- (TNF-α, IL-6, and IL-1β) and anti-inflammatory (TGF-β1, IL-4, and IL-10) cytokines in PMA-stimulated macrophages. As expected, the expression level of Nox1 (+2.05-folds, *p* < 0.01 vs. CTRL) and Nox2 (+3.74-folds, *p* < 0.001 vs. CTRL) mRNAs was significantly increased following PMA treatment (Figure 4A,B).

Among the three carnosine concentrations used in our study, the highest concentration (20 mM) was able to significantly decrease PMA-induced Nox1 activation (*p* < 0.05 vs. PMA), restoring values not significantly different from those determined in controls. Both 10 and 20 mM carnosine effectively decreased PMA-induced overexpression of Nox2 (*p* < 0.01 vs. PMA for both of them). The treatment of RAW 264.7 macrophages with PMA, in presence or absence of increasing concentration of carnosine did not significantly affect mRNA iNOS expression (Figure 4C).

**Figure 5:**
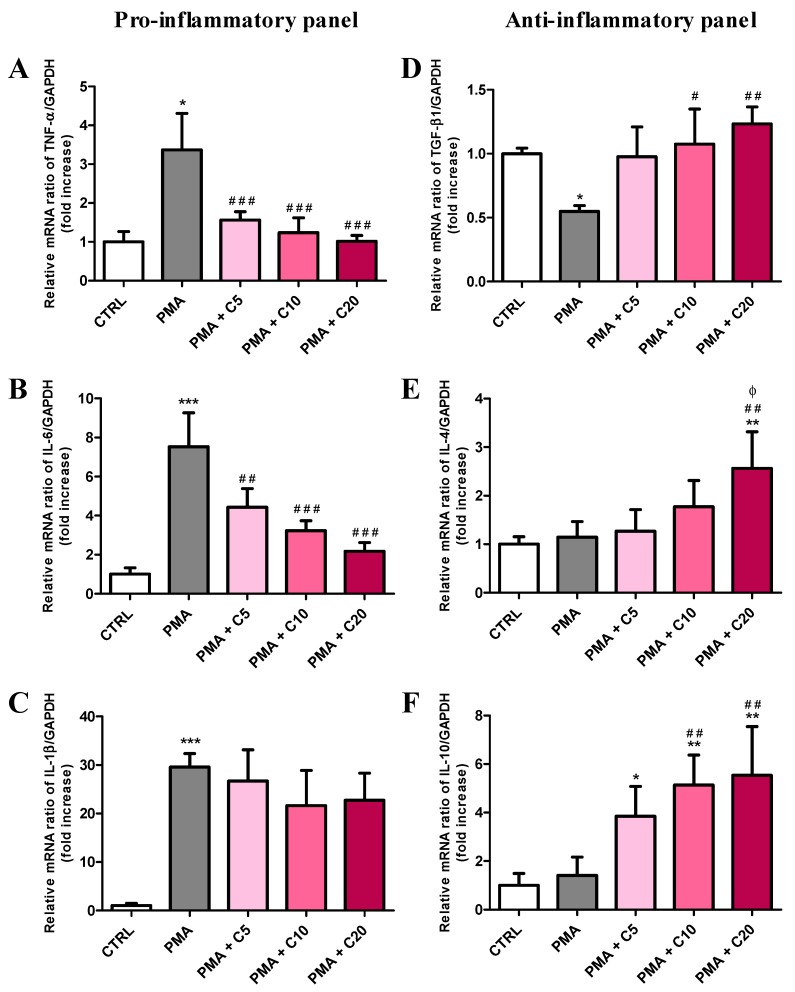
Measurement of (**A**) TNF-α, (**B**) IL-6, (**C**) IL-1β, (**D**) TGF-β1, (**E**) IL-4, and (F) IL-10mRNA expression levels (qRT-PCR) in resting (control) macrophages and in macrophages stimulated with PMA and SOD inhibitors, in absence or presence of increasing concentrations (5, 10, or 20) of carnosine. GAPDH-mRNA and NTC reactions were used as internal and negative controls, respectively, as described in Figure 4. Values are means ± SD of three to seven independent experiments. * *p* < 0.05 vs. CTRL; ** *p* < 0.01 vs. CTRL; *** *p* < 0.001 vs. CTRL; ^#^ *p* < 0.05 vs. PMA; ^##^ *p* < 0.01 vs. PMA; ^###^ *p* < 0.001 vs. PMA; ^ϕ^ *p* < 0.05 vs. PMA + C5.

Figure 5 shows the expression of genes related to the production of pro- (A–C) and anti-inflammatory (D–F) cytokines in resting (control) RAW 264.7 macrophages and in macrophages stimulated with PMA and SOD inhibitors, in absence or presence of increasing concentrations (5, 10, or 20) of carnosine.

Compared to control cells (Figure 5A–C), PMA treatment significantly increased the expression levels of TNF-α (+3.36-folds, *p* < 0.05), IL-6 (+7.51-folds, *p* < 0.001), and IL-1β (+29.56-folds, *p* < 0.001). Carnosine decreased TNF-α levels in a concentration-dependent manner (*p* < 0.001 vs. PMA for all carnosine concentrations) up to values equaling those found in controls, when carnosine was used at 10 or 20 mM. A similar trend was observed in the case of IL-6 (*p* < 0.01 vs. PMA for PMA + C5 and *p* < 0.001 vs. PMA for PMA + C10 or C20), although IL-6 expression was higher than control even in presence of 20 mM carnosine. Unlike TNF-α and IL-6, carnosine treatment did not significantly decrease the remarkable increased in IL-1β mRNA expression induced by PMA (*p* < 0.001 vs. CTRL). Significant decrease in the expression levels of TGF-β1 was recorded after the challenge of macrophages with PMA (−0.43-folds, *p* < 0.05 vs. CTRL)) (Figure 5D). Compared to control cells, PMA stimulation did not affect the production of IL-4 and IL-10 (Figure 5E–F). Each of the three carnosine treatments (5, 10, and 20 mM) was able to restore TGF-β1 expression (Figure 5D), whilst only the 20 mM concentration (Figure 5E) significantly increased IL-4 expression (*p* < 0.01 vs CTRL; *p* < 0.01 vs. PMA; *p* < 0.05 vs. PMA + C5). It is also worth underlining that 10 and 20 mM carnosine significantly increased IL-10 expression (Figure 5F) notwithstanding the stimulation with PMA (*p* < 0.01 vs. PMA or vs. CTRL).

### 3.4. Carnosine Effects on PMA-Induced Akt Phosphorylation in Cultured Macrophages

**Figure 6:**
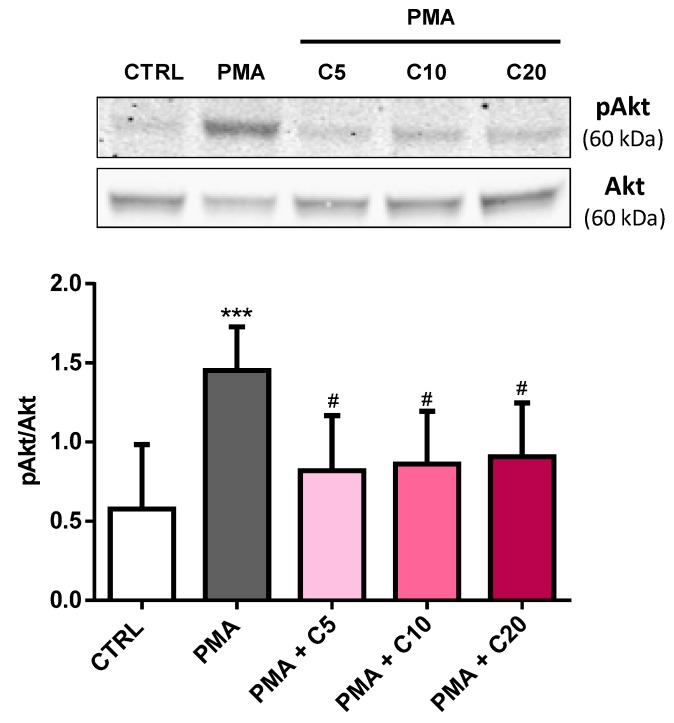
Carnosine partially reduced Akt phosphorylation levels PMA-induced in macrophages. Representative immunoblots of phosphorylated Akt (p(ser473)Akt) and total Akt (Akt) in total protein extracts from resting (control) macrophages and macrophages stimulated with PMA and SOD inhibitors, in absence or presence of increasing concentrations (5, 10, or 20) of carnosine. Histograms refer to the means ± SD of five independent experiments. The densitometric values of pAkt bands were normalized against Akt. *** *p* < 0.001 vs. CTRL; ^#^ *p* < 0.05 vs. PMA.

To address whether the ability to decrease oxidative stress and inflammation was also connected to other beneficial molecular phenomena, the effect of carnosine pre-treatment on Akt phosphorylation, Nrf2, and HO-1 was studied in PMA-stimulated macrophages. As showed in Figure 6, the stimulation of macrophages with PMA and SOD inhibitors strongly increased the activation of Akt as assessed by immunoblot analysis of phosphorylated-Akt (p(ser473)Akt) (from ~0.6 to ~1.45 pAkt/Akt; *p* < 0.001 vs. CTRL).

Carnosine was able to significantly attenuate this phenomenon (from ~1.45 to ~0.85 pAkt/Akt, *p* < 0.05) but no differences were observed between the three concentrations tested.

In the case of Nrf2 (total and nuclear) and HO-1 protein analysis, as it was expected considering the short duration of the stimulation time (60 min), no differences were observed among all the different experimental conditions (Figure S2).

## 4. Discussion

The connections among oxidative/nitrosative stress, NADPH oxidase and NOS activity, inflammation, and damages to biomolecules have well been characterized in various experimental studies, using macrophages as the typical cell line actively participating to all the aforementioned phenomena [56,57,58,59].

In the present study, using the macrophage RAW 264.7 cell line, we demonstrated that carnosine is a compound effectively interfering with ROS generation, NADPH oxidase expression, and the production of pro- and anti-inflammatory mediators. 

We initially explored the production of O_2_^−^• induced by PMA and SOD inhibitors as well as the antioxidant capacity of carnosine (or its methylated analogue anserine), in the RAW 264.7 macrophage cell line, representing an established in vitro experimental model for studying oxidative stress [1,44,45,46,47]. Among the various protocols tested, we selected an acute stimulation with PMA (60 min) (Figure 1A) giving the best combination of O_2_^−^• production and cell viability. To test carnosine activity, cells were pre-treated for 24 h with this dipeptide since, according to our previous results, the uptake of carnosine by RAW 264.7 macrophages increases proportionally during this time window, and it is ~3 times higher under stressing conditions [60]. In the present experiments, we found that carnosine was able to decrease the O_2_^−^• production PMA-induced in a concentration-dependent manner, with maximal effect observed when macrophage cells were pre-treated with 20 mM carnosine (Figure 1B). This specific carnosine feature is likely to be of great benefit in the case of cardiovascular disease, especially in the case of heart failure [61], hypertension and atherosclerosis [62], characterized by excessive production and/or inadequate removal of ROS, especially O_2_^−^• [63]. Our findings are in agreement with the previously shown carnosine antioxidant activity [38] and its ability to directly interact with different reactive species, decreasing their bioavailability [41,64]. In order to prove that the observed decrease in O_2_^−^• levels was in part attributable to the H atom of the secondary amine of the imidazole ring of carnosine (easily removable as H^.^ during ROS-mediated oxidation reactions), macrophages were pre-treated with anserine rather than with carnosine. In line with the well-known antioxidant properties of histidine [65,66], anserine, presenting a methyl group replacing H atom of the secondary amine of the histidine ring, was always less effective than carnosine in reducing O_2_^−^• levels at each concentration tested (Figure 1C). The stronger carnosine activity can in part explain why during clinical studies investigating the potential beneficial effects of dietary anserine/carnosine mixture supplementation on elderly people, anserine is always in excess with respect to carnosine (from 2:1 to 3:1) [67,68,69].

A lot of in vitro and in vivo studies linking carnosine antioxidant activity with its protective features are available [70,71]. In one of them, Tsai and co-workers showed the ability of this dipeptide to protect mice treated with 1-methyl-4-phenyl-1,2,3,6-tetrahydropyridine (MPTP) significantly attenuating glutathione loss, retaining the activity of glutathione peroxidase and SOD enzymes, diminishing oxidative stress, lowering pro-inflammatory cytokines and nitrite levels, and finally suppressing iNOS activity [72].

Under the experimental conditions to stress RAW 264.7 cells, PMA caused a significant decrease in the NAD^+^/NADH ratio and an increase in the NADP^+^/NADPH ratio (Figure 3B), demonstrating profound changes in the cell oxido-reductive state. While the change in the NAD^+^/NADH ratio was of relatively minor entity, confirming the PMA ability to increase the glycolytic rate [73], the almost 3 times increase in the NADP^+^/NADPH ratio strongly suggests the activation of NADPH oxidase during PMA-mediated oxidative burst [74].

In these experiments, only pre-treatment with 20 mM carnosine before the challenge with PMA significantly modified the cell energy state. Contrary to previous results obtained in cultured astrocytes, showing remarkable decrease in ATP induced by extracellular carnosine [75], we here found that the highest dose of carnosine tested not only caused a significant increase in ATP, but also a significant increase in GTP. According to our results, the raise in ATP might be related to increased mitochondrial oxidative phosphorylation linked to an augmented electron transfer chain and tricarboxylic acid activity [76,77]. Data showing a dose dependent increase in the carnosine-mediated NAD^+^/NADH ratio (particularly evident when 20 mM carnosine was used) supported this hypothesis. It is also worth underlining that carnosine dose-dependently decreased the NADP^+^/NADPH ratio, strongly indicating the decrease in the activity in the main actor (NADPH oxidase) responsible for ROS production and confirming that the improvement of cell bioenergetics has the inhibitory effects of a PMA-induced oxidative burst [78]. This ability of carnosine to modulate energy metabolism, with specific reference to ATP, could help reinforce the idea of its use in the treatment or prevention of diverse age-related conditions characterized by energy dysmetabolism [79].

According with the results showed in Figure 1B and with what was aforementioned, carnosine down-regulated the expression of both Nox1 and Nox2 enzymes, especially at the highest concentration (20 mM) (Figure 4A,B). These results are in agreement with the ability of this dipeptide to decrease oxidative stress-related enzymes, protecting microglial cells challenged with the oligomeric form of amyloid-β peptide [43]. A completely different scenario was observed when we investigated the effect of carnosine on pro- and anti-inflammatory cytokine genes expression in stimulated macrophages. In fact, on one hand the pre-treatment with carnosine was able to significantly (*p* < 0.001) down-regulate the expression of TNF-α and IL-6 mRNAs (Figure 5A,B); on the other hand, carnosine up-regulated the expression of IL-4 and IL-10 mRNAs (Figure 5E,F), also rescuing the TGF-β1 expression level to the values detected in resting cells, which was significantly higher than that of PMA alone (Figure 5D). These results point to an important role of carnosine in counteracting inflammatory phenomena promoting the transition in macrophages from a pro-inflammatory to an anti-inflammatory phenotype [80]. This “macrophage transition” plays a fundamental role in many diseases; in fact, the ratio of pro-inflammatory/anti-inflammatory macrophages, highly regulated in normal tissues, increases during diseases [80,81]. Therefore, the ability of carnosine to push towards an anti-inflammatory macrophage phenotype could, in part, make the difference between health and a pathological state.

During the last decade, the therapeutic potential of TGF-β1 in diseases characterized by oxidative stress and inflammation such as AD has emerged [82]. Carnosine in our experimental model induced a robust TGF-β1 overexpression. It has been shown that TGF-β1 administration protects rats against neuroinflammation and neurodegeneration Aβ-induced [82,83,84,85], while TGF-β1 knockout mice die from multifocal inflammation and autoimmune disorders in internal organs [86,87], highlighting the protective role of this multifunctional cytokine in protecting against inflammatory events. The ability of carnosine in counteracting the activation of Akt, enhanced in different cell types under stress conditions [48,88] and connected to the production of pro-inflammatory cytokines in macrophages [48], confirmed previous results obtained under different experimental conditions [48,89,90,91].

Our study produced robust evidences that carnosine effectively inhibits pro-oxidant and pro-inflammatory conditions in macrophages. Results demonstrated that the effects of carnosine are not only connected to the modulation of canonical pathways and activities causing oxidative/nitrosative stress and inflammation, but also that they are strictly linked to the capacity of this molecule to interact positively with the cell bioenergetics. Since the number of pathological conditions causing mitochondrial dysfunction and energy deficit are continuously increasing [92], it appears that the data showing the unexpected beneficial effects of carnosine on energy metabolism, related to improved mitochondrial oxidative phosphorylation and electron transport chain, looks particularly relevant in light of the potential application of this dipeptide in the treatment of pathologies such as neurodegenerative disorders.

## 5. Conclusions

In the present study we provided evidence that carnosine is able to reduce oxidative stress and inflammation, simultaneously restoring the parameters of cellular energy metabolism in activated macrophage cells. We reported for the first time that carnosine reduces PMA-induced oxidative stress in RAW 264.7 macrophage cells by decreasing the expression of Nox1 and Nox2 genes as well as the intracellular superoxide anion levels. In our experimental model, carnosine was also able to attenuate the PMA-induced Akt phosphorylation, down-regulate the expression of TNF-α and IL-6 mRNAs, simultaneously up-regulating the expression of the anti-inflammatory mediators IL-4 and IL-10, and rescuing TGF-β1 expression levels. Further, when used at the highest concentration, the antioxidant and anti-inflammatory effects of carnosine were accompanied by a generalized amelioration of the macrophage energy state, evaluated through the increase both in the total nucleoside triphosphate concentrations and the sum of the pool of intracellular nicotinic coenzymes. Finally, carnosine decreases the NADP^+^/NADPH ratio in a concentration dependent manner, indicating a strong inhibitory effect of this molecule towards the main source of ROS in this cell line.

## Figures and Tables

**Table 1:** The list of primers used for quantitative real-time PCR (qRT-PCR).

Official Name ^#^	Official Symbol	Alternative Titles/Symbols	Detected Transcript	Amplicon Length	Cat. No. ^†^
nitric oxide synthase 2, inducible	Nos2	iNOS; Nos-2; Nos2a; i-NOS; NOS-II; MAC-NOS	NM_010927	118 bp	QT00100275
NADPH oxidase 1	Nox1	MOX1; NOH1; NOH-1; NOX1a; Nox-1; GP91-2; NOX1alpha	NM_172203 XM_006528515	180 bp	QT00140091
cytochrome b-245, beta polypeptide	Cybb	Cgd; Cyd; Nox2; C88302; gp91-1; gp91phox; CGD91-phox	NM_007807 XM_006527565	146 bp	QT00139797
interleukin 1 beta	Il1b	Il-1b; IL-1beta; IL-1β	NM_008361 XM_006498795	150 bp 682 bp	QT01048355
tumor necrosis factor	Tnf	DIF; Tnfa; TNF-a; TNFSF2; Tnlg1f; Tnfsf1a; TNFalpha; TNF-alpha; TNF-α	NM_013693 NM_001278601	112 bp 112 bp	QT00104006
interleukin 6	Il6	Il-6	NM_031168	128 bp	QT00098875
interleukin 4	Il4	Il-4; BSF-1	NM_021283	132 bp	QT02418311
interleukin 10	Il10	CSIF; Il-10	NM_010548	103 bp	QT00106169
transforming growth factor, beta 1	Tgfb1	Tgfb; Tgfb-1; TGFbeta1; TGF-beta1	NM_011577	145 bp	QT00145250
glyceraldehyde-3-phosphate dehydrogenase	Gapdh	Gapd	NM_008084 XM_001003314 XM_990238 NM_001289726	144 bp	QT01658692

^#^ [52]; ^†^ [53].

## Supplementary Materials

The following are available online at https://www.mdpi.com/2076-3921/8/8/281/s1, Figure S1: Experimental procedure for the determination of O_2_^−^• levels in RAW 264.7 macrophages using microchip electrophoresis with laser-induced fluorescence (ME-LIF) and MitoSOX Red probe; Figure S2: Representative immunoblots of (A) total Nrf2, (B) nuclear Nrf2, and (C) HO-1 in protein extracts from resting (control) macrophages and macrophages stimulated with PMA and SOD inhibitors, in absence or presence of increasing concentrations (5, 10, or 20) of carnosine. Histograms refer to the means ± SD of three independent experiments. The densitometric values of total Nrf2 and HO-1 bands were normalized against GAPDH. The densitometric values of nuclear Nrf2 were normalized against Histone H3.
