# Supplementary material for: Carnosine Decreases PMA-Induced Oxidative Stress and Inflammation in Murine Macrophages
Source: Antioxidants (Basel). 2019 Aug 6;8(8):281. doi: 10.3390/antiox8080281 (PMC6720685; doi:10.3390/antiox8080281)
Supplement: Supplementary file 1 [file antioxidants-08-00281-s001.pdf]

Supplementary Figure legends

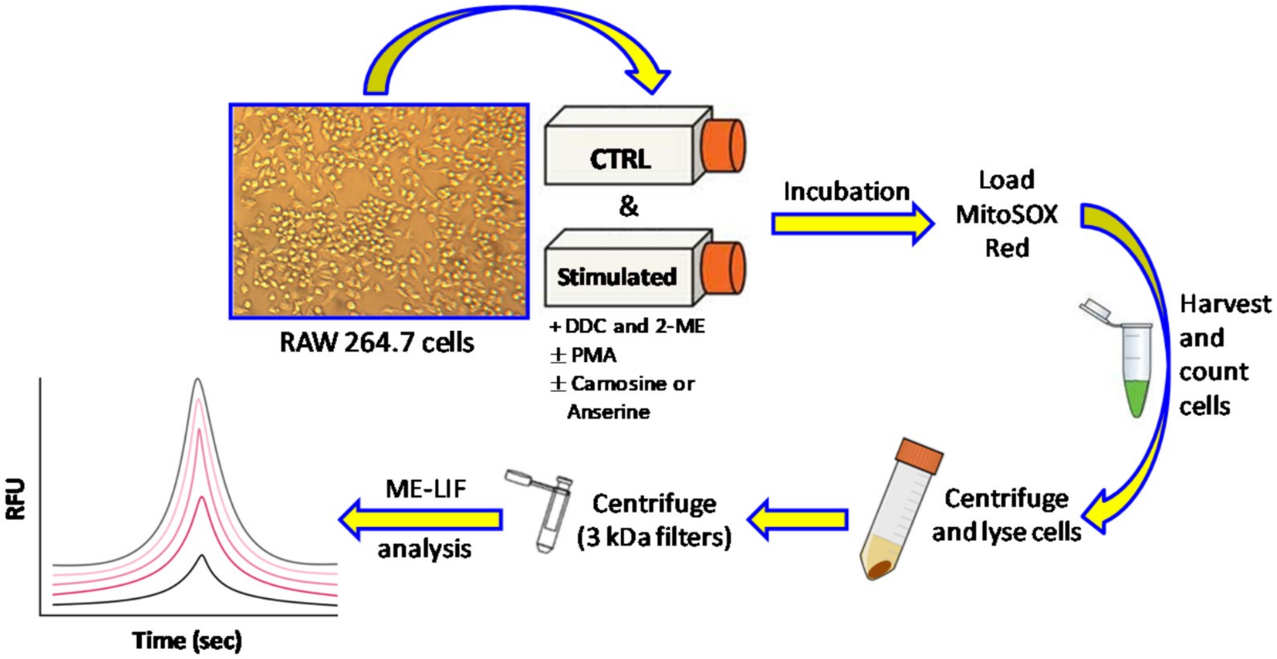

Figure S1. Experimental procedure for the determination of O_2^{-}• levels in RAW 264.7 macrophages using microchip electrophoresis with laser-induced fluorescence (ME-LIF) and MitoSOX Red probe.

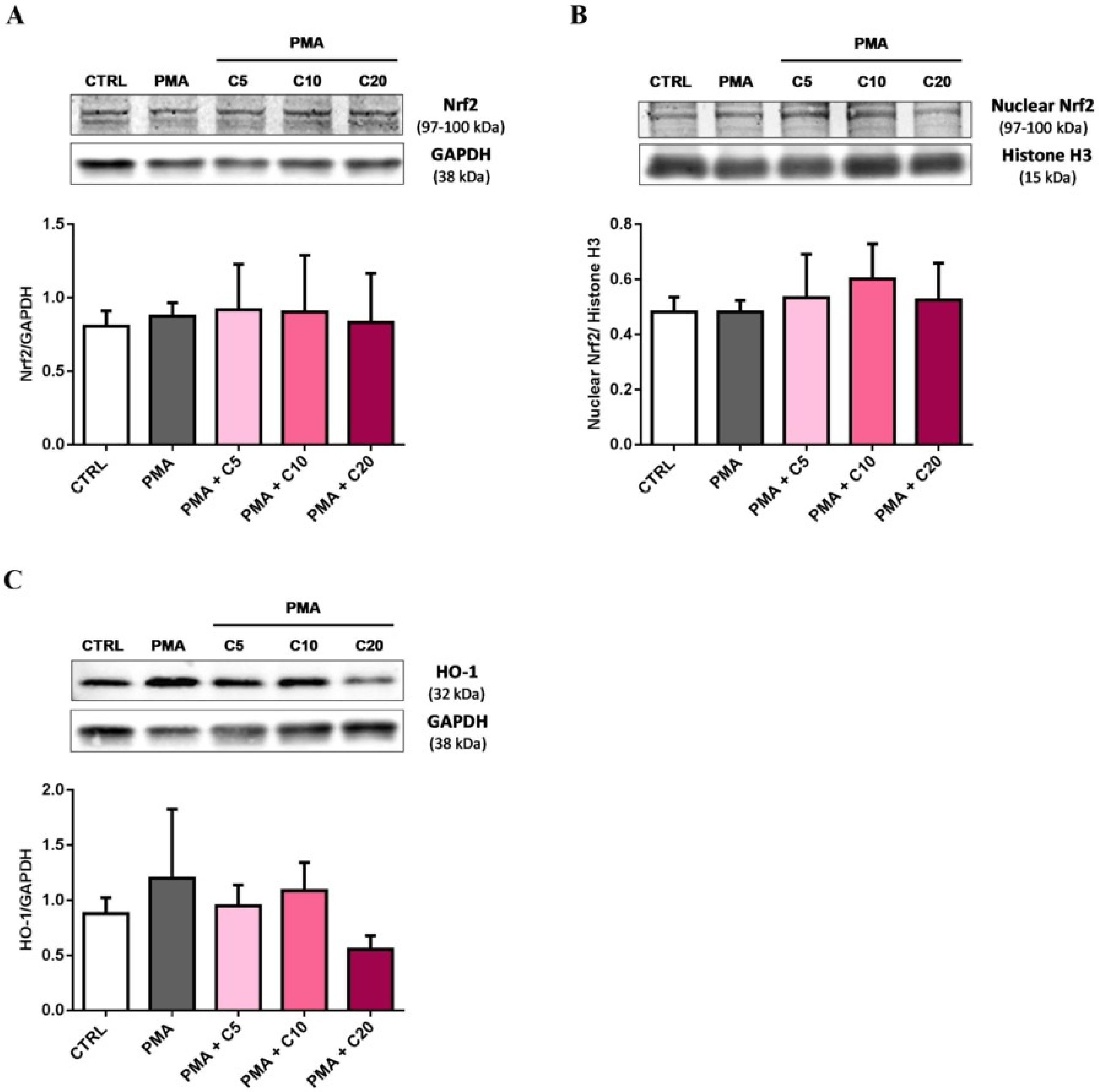

Figure S2. Representative immunoblots of (A) total Nrf2, (B) nuclear Nrf2, and (C) HO-1 in protein extracts from resting (control) macrophages and macrophages stimulated with PMA and SOD inhibitors, in absence or presence of increasing concentrations (5, 10, or 20) of carnosine. Histograms refer to the means ± SD of three independent experiments. The densitometric values of total Nrf2 and HO-1 bands were normalized against GAPDH. The densitometric values of nuclear Nrf2 were normalized against Histone H3.
